# Cardiac arrest following cardiac tamponade caused by mycosis fungoides malignant pericarditis

**DOI:** 10.1002/ccr3.5042

**Published:** 2021-11-19

**Authors:** Shuku Sato, Eri Tanaka

**Affiliations:** ^1^ Division of Hematology Shonan Kamakura General Hospital Kamakura Japan; ^2^ Division of Internal Medicine Hayama Heart Center Hayama Japan

**Keywords:** cardiac tamponade, mycosis fungoides

## Abstract

Mycosis fungoides is a low‐grade lymphoma, but on reaching the tumor stage, it can cause cardiac tamponade owing to epicardial infiltration. Myocardial infiltration, even in the absence of abnormal imaging findings, requires attention because it can lead to arrhythmia and cardiac arrest.

## Graphical abstract

Mycosis fungoides is an indolent lymphoma, but when it reaches the tumor stage, it can cause cardiac tamponade due to extracardiac invasion, which may lead to arrhythmia and sudden death due to myocardial invasion.

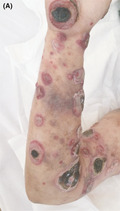

A 48‐year‐old man diagnosed with mycosis fungoides (MF), advanced to the tumor stage (Figure 1A), presented with dyspnea and leg edema. He was receiving gemcitabine at 1.2 g/m^2^ once weekly for 8 weeks. Echocardiography revealed cardiac tamponade caused by malignant pericarditis, and computed tomography (CT) revealed bilateral malignant pleural effusions (Figure 1B–D). However, echocardiography, CT, and magnetic resonance imaging showed no evidence of wall thickening or intramyocardial masses suggesting myocardial infiltration. We drained 2 L of pericardial fluid, which showed T cell clonality by Southern blot analysis while starting radiation therapy to the pericardium. The same findings were seen with the pleural effusion. Thereafter, he developed a complete atrioventricular block, followed by cardiac arrest. In this case, MF might have invaded the myocardium and disordered the impulse conduction system. Malignant lymphoma (ML) occasionally presents with cardiac involvement, but most patients have no suggestive symptoms, and diagnosis while alive is difficult.^1^ William et al. reported that out of 48 autopsies of ML cases with cardiac involvement, only 37% involved the epicardium only, while the rest involved the ventricular wall and epicardium.^2^ MF is a low‐grade lymphoma, but on reaching the tumor stage, it can cause cardiac tamponade owing to epicardial infiltration. Myocardial infiltration, even in the absence of abnormal imaging findings, requires attention because it can lead to arrhythmia and cardiac arrest.

**FIGURE 1:**
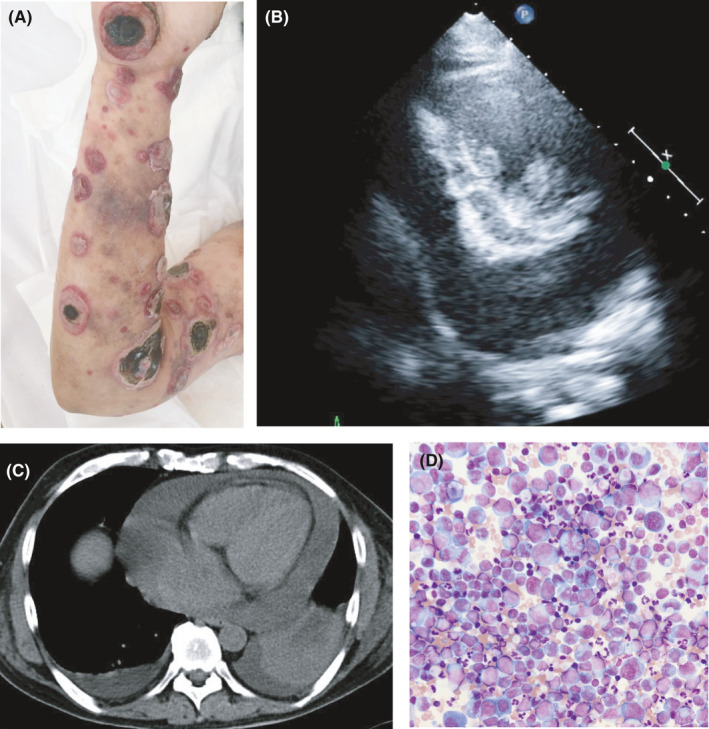
(A) Tumors due to MF were found in various parts of his extremities and trunk. The tumors were protuberant, 1–10 cm in size, and the aborted areas were necrotic. (B) Echocardiographic findings showed that the left ventricle was depressurized due to pericardial effusion. (C) Computed tomography findings showed pericardial and bilateral pleural effusions and pneumonia in the left lower lobe. (D) Cytology of the pericardial fluid showed malignant cells

## CONFLICT OF INTEREST

All authors declare no conflicts of interest.

## AUTHOR CONTRIBUTIONS

SS and ET participated in the management of this patient. SS prepared and edited this manuscript.

## ETHICAL APPROVAL

The present article has been approved by the Ethics Committee of Shonan Kamakura General Hospital, in accordance with the Declaration of Helsinki.

## CONSENT

Written informed consent was obtained from the patient.

## Data Availability

The data that support the findings of this study are available from the corresponding author upon reasonable request.
